# Metabolic Profiling as an Approach to Differentiate T-Cell Acute Lymphoblastic Leukemia Cell Lines Belonging to the Same Genetic Subgroup

**DOI:** 10.3390/ijms25073921

**Published:** 2024-03-31

**Authors:** Husam B. R. Alabed, Roberto Maria Pellegrino, Sandra Buratta, Anair Graciela Lema Fernandez, Roberta La Starza, Lorena Urbanelli, Cristina Mecucci, Carla Emiliani, Paolo Gorello

**Affiliations:** 1 Department of Chemistry, Biology and Biotechnology, University of Perugia, 06100 Perugia, Italyroberto.pellegrino@unipg.it (R.M.P.); sandra.buratta@unipg.it (S.B.); lorena.urbanelli@unipg.it (L.U.); 2 Centro di Eccellenza sui Materiali Innovativi Nanostrutturati (CEMIN), University of Perugia, Via del Giochetto, 06123 Perugia, Italy; 3 Hematology and Bone Marrow Transplantation Unit, Laboratory of Molecular Medicine (CREO), Department of Medicine and Surgery, University of Perugia, 06132 Perugia, Italy; anairgraciela.lemafernandez@unipg.it (A.G.L.F.); cristina.mecucci@unipg.it (C.M.)

**Keywords:** untargeted metabolomics, lipidomics, T-ALL, lymphoblastic leukemia, TAL/LMO

## Abstract

T-cell acute lymphoblastic leukemia (T-ALL) is an aggressive tumor mainly affecting children and adolescents. It is driven by multiple genetic mutations that together define the leukemic phenotype. Interestingly, based on genetic alterations and/or deregulated expression, at least six genetic subgroups have been recognized. The TAL/LMO subgroup is one of the most represented genetic subgroups, characterizing 30–45% of pediatric T-ALL cases. The study of lipid and metabolic profiles is increasingly recognized as a valuable tool for comprehending the development and progression of tumors. In this study, metabolic and lipidomic analysis via LC/MS have been carried out on four T-ALL cell lines belonging to the TAL/LMO subgroup (Jurkat, Molt-4, Molt-16, and CCRF-CEM) to identify new potential metabolic biomarkers and to provide a subclassification of T-ALL cell lines belonging to the same subgroup. A total of 343 metabolites were annotated, including 126 polar metabolites and 217 lipid molecules. The statistical analysis, for both metabolic and lipid profiles, shows significant differences and similarities among the four cell lines. The Molt-4 cell line is the most distant cell line and CCRF-CEM shows a high activity in specific pathways when compared to the other cell lines, while Molt-16 and Jurkat show a similar metabolic profile. Additionally, this study highlighted the pathways that differ in each cell line and the possible enzymes involved using bioinformatic tools, capable of predicting the pathways involved by studying the differences in the metabolic profiles. This experiment offers an approach to differentiate T-ALL cell lines and could open the way to verify and confirm the obtained results directly in patients.

## 1. Introduction

T-cell acute lymphoblastic leukemia (T-ALL) is a highly aggressive form of lymphoid tumor caused by the malignant transformation of T cells [1]. T-ALL represents approximately 15% of pediatric cases and 25% of adult cases among all lymphoblastic leukemia instances [2,3,4]. Patients show symptoms that are typically linked to anemia, thrombocytopenia, and neutropenia due to the infiltration of the bone marrow by tumor cells [5].

In recent years, the genetic basis of T-ALL has been largely explored [6,7,8,9]. Genomic studies have provided the genetic classification of T-ALL based on alterations/deregulated expression of transcription factors with pivotal roles in T-cell differentiation. In this context, at least six subgroups have been recognized. They are TAL/LMO, HOXA, TLX3, TLX1, NKX2-1/2-2, and BCL11B. In addition, abnormalities of other gene classes that control self-renewal capacity, cell cycle, proliferation, apoptosis, and/or differentiation cooperate in the leukemia phenotype [10,11].

While most efforts are directed toward the genetic characterization of tumors, it is becoming evident that the study of metabolic and lipid profiles provides additional insight into the pathogenesis and progression of tumors [12,13].

Metabolomics and lipidomics are emerging fields of biochemistry that study metabolic pathways and metabolic processes in a biological system [14,15,16]. Recently, liquid chromatography/mass spectrometry (LC/MS) has become the most used approach in laboratories to study metabolic and lipid profiles [17,18]. This analytical approach allows the generation of complex data matrices that can be studied and analyzed with several bioinformatics tools such as the MetaboAnalyst 5.0 web platform for statistical and pathway analysis [19], the OmicsNet web platform for biological networks [20], LipidOne for lipidomic data analysis [21], and Biopan for lipid metabolic pathways [22].

In the field of research, these methods offer priceless insights into biological pathways and processes, helping scientists better understand how diseases, genetic mutations, and the environment affect these pathways [23,24,25] and allowing them to find potential therapeutic targets or biomarkers [26,27,28,29]. In this context, it would be particularly interesting to deepen the metabolic and lipid studies of T-ALL to identify and highlight the significant differences between the different forms of leukemia. Such investigation could provide a deep understanding of these types of tumors, opening new research perspectives and potentially revealing important diagnostic and therapeutic information.

The TAL/LMO transcriptional complex subgroup constitutes a substantial proportion of T-ALL cases, comprising 30–45% of pediatric T-ALL cases and 10–15% of adult T-ALL cases [10]. In light of this, it would be interesting to investigate the metabolomic and lipidomic profiles of T-ALL tumor cells taken from multiple sources and belonging to this subgroup to study any metabolic similarity or differences inside the same subgroup. Four cell lines have been selected for this study: Jurkat, CCRF-CEM, MOLT-16, and MOLT-4. All of them belong to the TAL/LMO transcriptional complex subgroup and all represent in vitro models of acute lymphoblastic leukemia. By digging into the molecular phenotype of these cell lines, we aim to identify potential variations among them, possibly allowing for the classification and stratification of the different cell lines at the metabolic level, unraveling differences, or similarities, in the same genetic subgroup. This characterization could offer valuable insights for further subclassification of this type of leukemia, shedding light on statistically significant differences in metabolites and lipids, which can eventually be verified in clinical cases.

To achieve this aim, metabolomic and lipidomic analyses were conducted on the selected T-ALL cell lines. At the level of polar metabolites, statistical and pathway analyses were performed. Regarding the study of lipids, statistical analyses were conducted at three different levels: lipid classes, lipid molecular species, and lipid building blocks.

## 2. Results

### 2.1. Polar Metabolite Analysis

Untargeted metabolomics analysis allowed the annotation of 126 polar metabolites: complete information is listed in Table S1. The data were normalized via the median and Log transformation (base 10) and were scaled with Pareto scaling.

**Figure 1:**
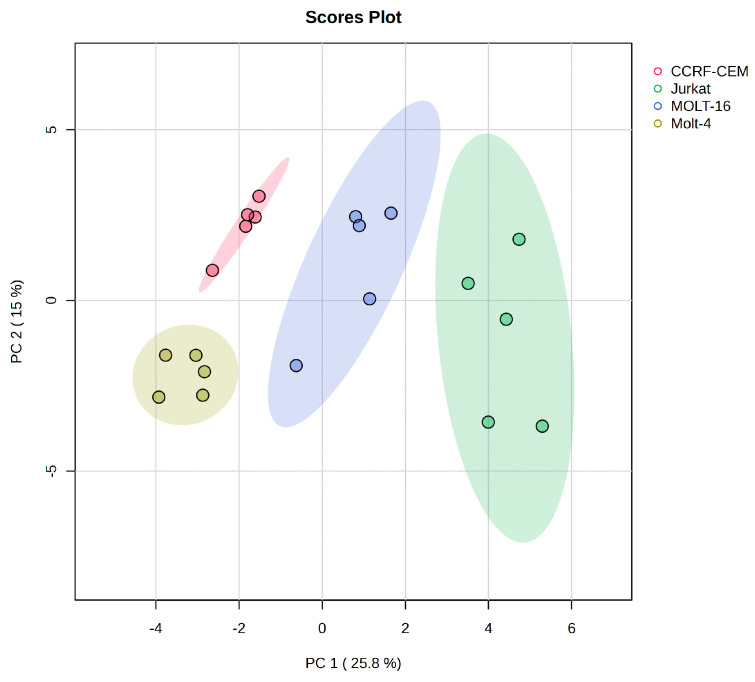
The figure shows the PCA scores plot; the ellipses enclose the scores inside a region with 95% confidence. The colors indicate the cell line type, and the symbols indicate the sample. The platform Metaboanalyst was used to generate these score plots.

Unsupervised principal component analysis (PCA) on the polar metabolite matrix was performed (Figure 1).

The PCA score of polar metabolites demonstrates clear separation among the different cell lines. In fact, the first component (that explains 25.8% of variance) divides the Jurkat and Molt-16 cell lines from the CCRF-CEM and Molt-4 cell lines, while the second component (15%) was able to separate between the CCRF-CEM and Molt-4 cell lines. In fact, according to the classification proposed by Burger, Renate, et al., which classify the cells based on their immunophenotype, the CCRF-CEM cell line is considered pre-T, while Jurkat and Molt-16 lines are classified as mature T and Molt-4 as cortical T [30]. This classification could provide an explanation regarding the marked separation of the CCRF-CEM cell line from the other three lines. Similarly, the separation of the cortical cell line, Molt-4, and ultimately the separation at the first component level of the two mature T-type cell lines, Jurkat and Molt-16, from the other two can be explained. Moreover, the cell lines are from different genders and ages (Jurkat: 14-year-old male, CCRF-CEM: 3-year-old girl, Molt-4: 19-year-old man, and Molt-16: 5-year-old girl); however, it appears that the sex and age of the patients from whom these lines were derived do not influence or explain the clustering shown in the PCA analysis.

**Figure 2:**
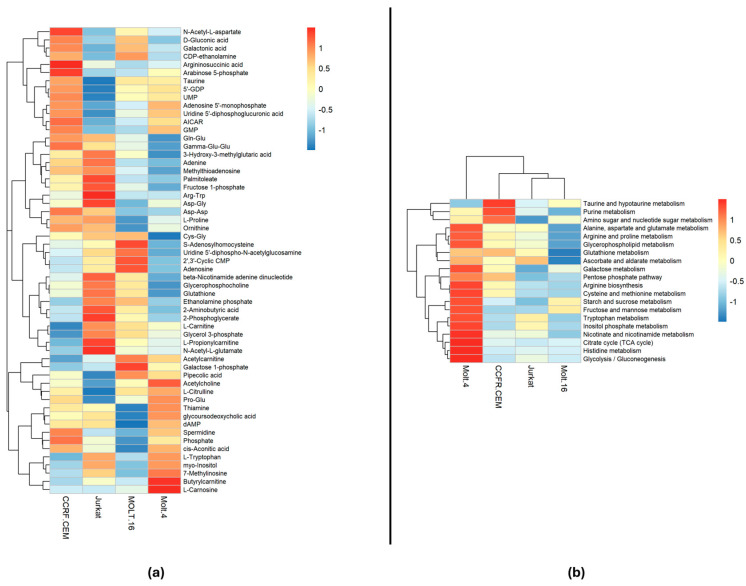
The heatmaps: (**a**) Displays the correlation of the 57 statistically significant polar metabolites within each cell line. The colors indicate the expression level of each individual metabolite in each cell line based on their relative abundance. (**b**) Presents the results of pathway analysis conducted on each cell line sample, comparing them to medium bootstrap samples. The colors represent the significance based on the −log(*p*-value) of each pathway in each cell line. The analysis employed the Euclidean correlation index and the complete clustering method.

A one-way ANOVA test with a q-value (FDR) cut-off of 0.05 was performed to highlight the statistically significant metabolites. The ANOVA test revealed 57 significant metabolites (see Table S2), while the remaining metabolites did not show significant differences among the cell lines. Notably, several amino acids and their derivatives, dipeptides, and other metabolites vary among the different groups (Figure 2a), suggesting the presence of variations affecting cellular pathways. Most of these metabolites are involved in at least one metabolic pathway (See Table S3).

Pathway analysis was conducted on the entire polar metabolite matrix in order to highlight pathway variations in each cell line. The results were visualized using a heatmap to show the significance intensity of each pathway in each cell line (Figure 2b). Pathways with an impact of less than 0.1 were excluded.

As indicated in Figure 2b, different metabolic pathways exhibit statistical differences among specific cell lines. Notably, Molt-4 appears to be the most distinct cell line based on its metabolic profile. Several pathways, including tryptophan metabolism, show statistically significant differences in Molt-4 compared to the other cell lines. This is supported by the elevated abundance of tryptophan in Molt-4 cells when compared to the other lines (Figure 2a). Additionally, glutathione metabolism in Molt-4 varies significantly, attributed to the lower abundance of glutathione and Cys-Gly in Molt-4 compared to the other three cell lines. Arginine biosynthesis also appears to be decreased in Molt-4, potentially due to the lower abundance of N-(L-Arginino) succinate and ornithine. Inositol phosphate metabolism is another pathway exhibiting variations in Molt-4, likely linked to the higher abundance of myo-inositol in Molt-4 cells compared to the other lines.

The CCRF-CEM cell line emerges as the second most distinct based on its metabolic profile. Differences in multiple metabolic pathways, including purine metabolism, are evident, with CCRF-CEM exhibiting higher levels compared to other cell lines. This is supported by the elevated presence of metabolites like GDP, adenosine, AMP, GMP, and adenine, which also play roles in cellular signaling. Taurine and hypotaurine metabolism show a similar pattern, with CCRF-CEM displaying higher levels, possibly due to increased taurine abundance. Similar to Molt-4, CCRF-CEM also exhibits significant variation in glutathione metabolism, suggesting potential differences in oxidative stress response compared to Jurkat and Molt-16. Moreover, metabolites such as acetylcholine vary in different ways in these cell lines; in fact, Molt-4 demonstrates a high abundance of it; on the other hand, Molt-16 and CCRF-CEM demonstrate similar abundance, while Jurkat demonstrates a low abundance of this metabolite.

On the other hand, Molt-16 and Jurkat demonstrate similar metabolic profiles, with minor differences compared to the other two cell lines.

**Figure 3:**
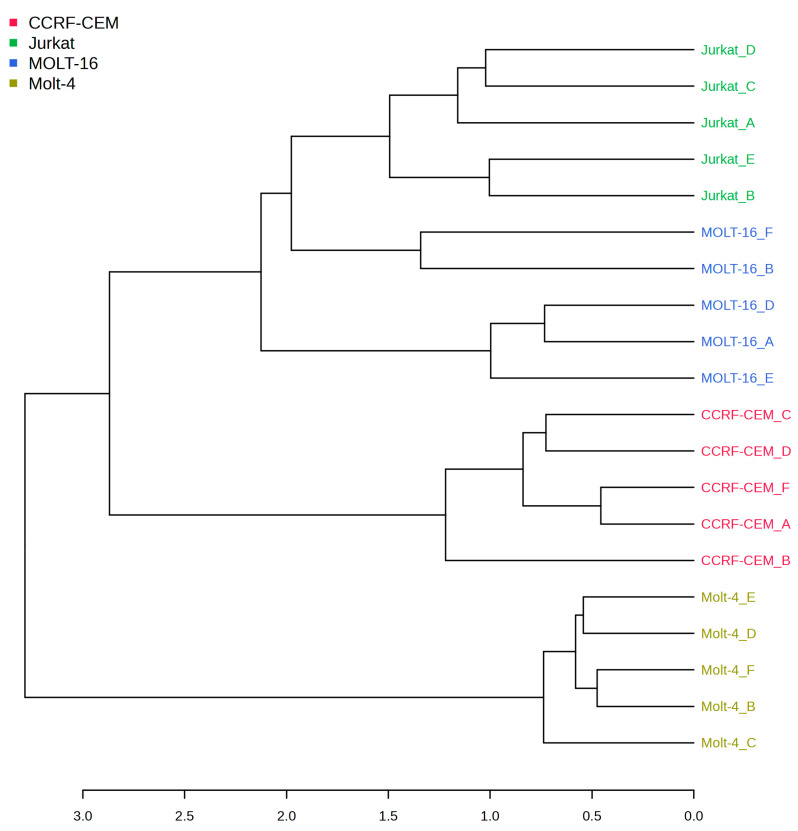
Hierarchical clustering dendrogram. The numbers below are the distances calculated with the Pearson algorithm. The tree was created by using the distance measure of the Pearson correlation and complete clustering algorithm.

Finally, a cluster analysis was performed based on all polar metabolites detected with the Dendrogram function (see Figure 3).

Hierarchical cluster analysis revealed clear segregation of all the analyzed cell lines from the Molt-4 (Figure 3) cell line. In fact, this result could indicate that Molt-4 has an overall different metabolic profile when compared to the other three. The remaining cell lines can be separated into two sub-branches: one containing Molt-16 and Jurkat, which mostly demonstrate a similar metabolic profile, and the second one containing the CCRF-CEM cell line.

### 2.2. Lipid Profile Analysis

After lipidomic workflow, a data matrix was obtained containing qualitative and semiquantitative information on 217 lipid molecular species divided into 19 classes (see Table S4). For a clearer and more detailed lipidomic analysis, qualitative and semiquantitative differences within lipid classes will be studied at first, then among molecular species, and finally among lipid building blocks.

#### 2.2.1. Analysis at the Level of Lipid Classes

**Figure 4:**
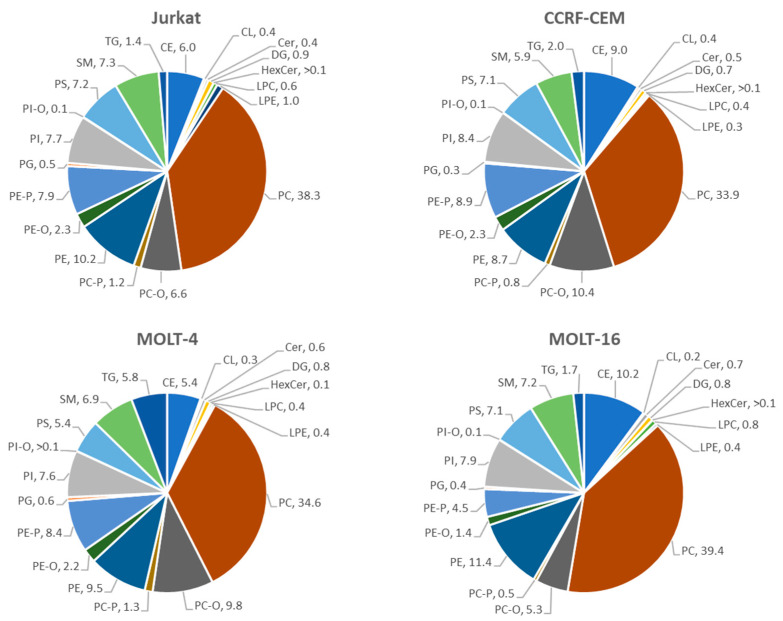
Pie chart: the figure shows the percentages of lipid classes referred to each cell line. Excel-Microsoft office 365 (version 2402) was used to create the charts after obtaining the percentages table by using an R script on the lipid data matrix. Every color represents one of the lipid classes.

The results at the level of lipid classes are expressed as the average and standard error. The *p*-value of the comparison among these four cell lines was calculated using one-way ANOVA. The results are shown in Table 1. We also calculated the percentage of lipid classes among each cell line analyzed (see Figure 4).

As indicated in Table 1, a significant difference appears in the total lipid content among the cell lines, especially between Jurkat and CCRF-CEM on the one hand and Molt-4 and Molt-16 on the other. Molt-4 exhibits the highest total lipid amount among the cell lines. Moreover, there are statistically significant differences in the amounts of specific lipid classes, including PC, PE, SM, TG, PC-O, PE-P, and others. In fact, both Molt-16 and Molt-4 demonstrate higher levels of PC and PE in comparison to the Jurkat and CCRF-CEM cell lines. Molt-4 also displays a remarkable quantity of TG compared to the other cell lines. Interestingly, Molt-16 presents a lower concentration of ether-linked lipids such as PE-P, PE-O, and PC-P when compared to the other cell lines.

In Figure 4, the percentage of each lipid class relative to the total lipids within each cell line is represented. The most represented lipid classes within all four cell lines are the phospholipids (PC, PE, PI, PG, PS, PE-O, PE-P, PC-O, PC-P, PI-O LPC, LPE, and SM) representing 90.9% in Jurkat, 87.4% in CCRF-CEM, 87.0% in Molt-4, and 86.2% in Molt-16. Notably, most lipids are distributed similarly among the cell lines and represent the majority of lipids inside them (PC, PE, PI, PG, PS, PE-O, PI-O, and SM). However, some lipid classes vary among the cell lines, as observed in Figure 4. Specifically, Molt-16 exhibits a relatively different distribution of certain lipid classes when compared to other cell lines, such as CE (10.2%), compared to Jurkat (6.0%) and Molt-4 (5.4%); PC-O (5.3%) compared to CCRF-CEM (10.4%), Jurkat (6.6%), and Molt-4 (9.8%); and PE-P (4.5%) compared to CCRF-CEM (8.9%), Jurkat (7.9%), and Molt-4 (8.4%). On the other hand, in Molt-4, the TG lipid class is uniquely distributed, representing 5.8% of total lipids and showing a higher percentage of this lipid class compared to Jurkat (1.4%), Molt-16 (1.7%), and CCRF-CEM (2.0%).

In this context, few studies on the lipid composition of T cells have been reported in the literature, which were mainly interested in T cells isolated from blood rather than immortalized cell lines or tumor models. A study from Alarcon-Barrera and colleagues on the lipid composition of CD4+ T cells [31] showed that the percentage of PC within these cells is 30% of lipids, while that of PE and SM is 20% and 30%, respectively. A subsequent study concerning differences in the lipid composition of the plasma membranes of T cells and B cells [32] showed that the percentages of PC within the cell membrane of T cells are about 65%, for PE the percentage is 8.6%, and for SM the percentage is 15.7%. Although these different results are due to noncomparable techniques, they provide indications about the most represented lipid classes within this cell type, which are precisely PC, PE, and SM. Our data confirmed this lipid composition on T-cell line models, and our matrix underlines the significant presence of other important lipid classes such as ether-linked PC, ether-linked PE, PI, and PS.

#### 2.2.2. Analysis at the Level of Lipid Molecular Species

The one-way ANOVA test revealed 170 significant lipid species (Table S5); it also includes the Tukey’s test.

**Figure 5:**
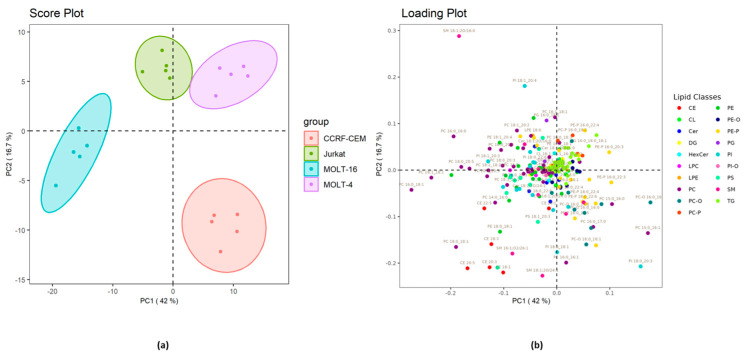
(**a**) PCA score plot of the lipid molecular species from the four cell lines. The ellipses enclose the scores inside a region with 95% confidence. (**b**) Loading plot of the lipid molecular species which contribute to the separation among the cell lines. Before the analysis, data matrix was normalized and pareto scaling was applied.

The lipidomic data matrix was subjected to principal component analysis (PCA). The results are illustrated in Figure 5.

The principal component analysis in Figure 5a highlights a clear separation between the four cell lines, both in the first component (42% of explained variance), between Molt-16 and Jurkat on the one hand and Molt-4 and CCRF-CEM on the other, and in the second component (16.7% of explained variance), between Molt-4 and Jurkat on one side and Molt-16 and CCRF-CEM on the other. These results are very similar to the results of the PCA on the polar metabolites (Figure 1). Observing Figure 5b, it is possible to note the lipid molecular species that contribute the most to the separation and clustering; in fact, most of these molecules are PC, SM, PE, PI, and CE.

**Figure 6:**
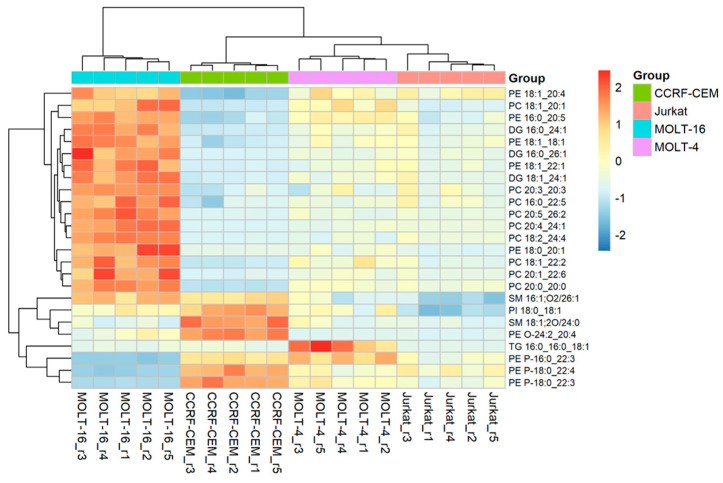
Heatmap: The figure shows the correlation of the 25 most significant lipid molecular species within each sample. The color shows the level of expression of each individual species in each sample based on their concentrations. The analysis was performed using the Pearson correlation index and the complete clustering method.

Heatmap visualization was applied on the lipidomic data matrix to study the quantitative differences among the single lipids inside the different cell lines. The results shown in Figure 6 highlight the main lipid molecular species with differences in expression.

It is worth mentioning that some molecular species of the PE P lipid class, such as PE P-16:0_22:3, PE P-18:0_22:4, and PE P-18:0_22:3, are present in concentrations relatively high in the CCRF-CEM cell line compared to the other lines. On the contrary, relatively low concentrations of these three lipids are found in the Molt-16 line, while the other two cell lines show average concentrations compared to CCRF-CEM. On the other side, numerous molecular species belonging to the PE and PC lipid classes are present in relatively high concentrations in the Molt-16 line, whereas relatively low concentrations are found in the CCRF-CEM line and relatively medium concentrations in the Jurkat and Molt-4 lines. Furthermore, some molecular species, such as SM 16:1;O2/26:1, SM 18:1;O2/24:0, PI 18:0_18:1, and PE O-24:2_20:4, show relatively high concentrations in the CCRF-CEM line and relatively low concentrations in the other cell lines. These results at the two levels confirm that the lipidic profile of Molt-16 is highly different from the other cell lines, especially CCRF-CEM, while Jurkat and Molt-4 demonstrate a similar lipid profile.

#### 2.2.3. Analysis of Lipid Building Blocks

**Figure 7:**
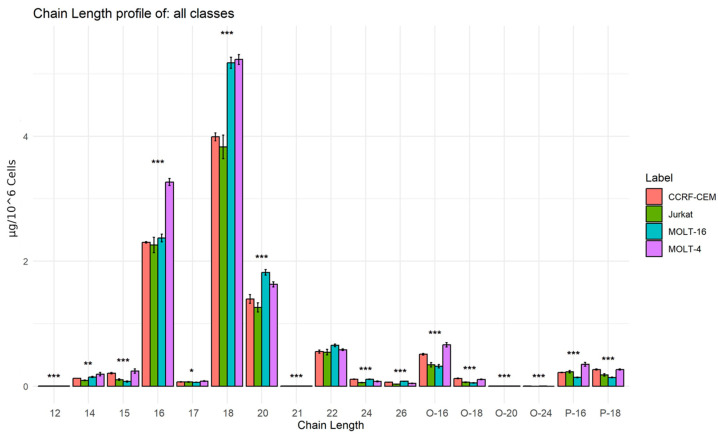
Bar graph: The figure shows the average concentrations (ug/10^6^ of cells) of the chains with different lengths. Experimental error bar (n = 5) and ANOVA significance asterisks are represented (*** indicates *p*-value < 0.001, ** indicates *p*-value < 0.01, and * indicates *p*-value < 0.05). This graph was made with R program (R version 4.2.3 (2023-03-15 ucrt)).

Ultimately, we further analyzed the composition of the building blocks in the context of lipid molecular species. Specifically, we analyzed the lengths of the chains and the amount of unsaturation of the fatty acids that make up the various lipids. Figure 7 shows the differences in the concentrations of chains with different lengths (with different numbers of carbon atoms).

The bar graph in Figure 7 shows that the most abundant chains are those composed of 16, 18, 20, and 22 carbon atoms. It is interesting to note that most of the chain lengths are statistically different: particularly, 18 and 20 carbon-atom chains are highly present in Molt-4 and Molt-16. Chains with 16 carbon atoms are predominantly present in Molt-4. Furthermore, a particular abundance of ether chains with 16 and 18 carbon atoms is observed, which are mostly present in the two cell lines Molt-4 and CCRF-CEM.

Regarding the unsaturation in the fatty acids of lipids, ratios of saturated fatty acids (SFA), monounsaturated fatty acids (MUFA), and polyunsaturated fatty acids (PUFA) were calculated based on the average amount (in µg/10^cells) of fatty acids with different types of saturation and unsaturation within the lipid species. Table 2 illustrates the results.

As shown in Table 2, the SFA/MUFA ratio in all four cell lines is less than one, indicating a lower quantity of saturated fatty acids compared to monounsaturated fatty acids inside the cells. Conversely, the SFA/PUFA ratios are all higher than 1, signifying a greater presence of saturated fatty acids than polyunsaturated fatty acids inside the cells. However, differences in the ratios among the cell lines do not reach significance. Nevertheless, the lower ratios in the Molt-16 cell line could suggest a higher activity in pathways involving the desaturation of fatty acids compared to the other cell lines.

## 3. Discussion

Statistical analyses of the relative abundances of polar metabolites confirm the presence of significant differences in the metabolism of the four T-ALL analyzed cell lines. The results of principal component analysis (PCA) applied to polar metabolite and lipid matrices showed that differences in metabolic and lipid profiles are able to cluster cell lines according to their maturation status. Additionally, specific pathways emerge that vary among them, such as tryptophan metabolism, glutathione metabolism, arginine biosynthesis, purine metabolism, and others. The analysis showed a higher abundance of tryptophan in Molt-4 when compared to the other cell lines, suggesting a different metabolic behavior for this amino acid in Molt-4. The catabolism of this amino acid is generally correlated with peripheral immune tolerance [33]. Additionally, Molt-4 showed a low abundance of glutathione and other metabolites involved in its metabolism, indicating a different oxidative stress activity. Enzymes such as glutathione synthetase and glutamate cysteine ligase, which contribute to glutathione metabolism [34], may have a lower activity in Molt-4 when compared to the other cell lines. Studies have shown that this pathway is closely related to tumors; in fact, high levels of glutathione promote tumor progression and increased metastasis [35].

The pathway analysis has also demonstrated lower arginine biosynthesis in Molt-4 in comparison to the other cell lines. This result could also indicate a variation in the activity of enzymes involved in this pathway, such as ornithine transcarbamylase and argininosuccinate synthase 1. Arginine biosynthesis is one of the most important and sensitive pathways in cancer cells, regulating cell death [36]. The identified differences within these T-ALL cell lines highlight the possibility to further investigate their sensitivity to new personalized therapeutic approaches.

Our pathway analysis demonstrated higher abundance in specific metabolites related to purine metabolism in the CCRF-CEM cell line, suggesting its higher activity in CCR-CEM. Moreover, enzymes such as guanylate kinase 1, phosphodiesterase 10A, and adenosine kinase play key roles in purine metabolism and may be involved in the identified deregulation. Numerous studies have demonstrated the importance of this pathway in cancer biology; indeed, purine metabolism appears to be increased in tumors because purine nucleotide metabolism is crucial for cancer cell proliferation. Accordingly, this pathway is therefore one of the targets for cancer therapies including some types of leukemia [37,38,39,40,41].

These results, at the level of polar metabolites, highlight potential biomarkers and distinct behaviors for some cell line. For instance, purine metabolism is distinguishable in CCRF-CEM, while the other cell lines exhibit similar characteristics. Moreover, Molt-4 exhibits a unique behavior in various metabolic pathways, particularly in arginine biosynthesis, the metabolism of glutathione, and tryptophan. The pathway analysis has also demonstrated a comparable metabolic profile in Jurkat and Molt-16.

The results of our hierarchical cluster analysis reveal a significant finding: the Molt-4 cell line exhibits the most distinct metabolic phenotype compared to the other three cell lines. It is noteworthy to highlight that the metabolic behavior of CCRF-CEM is also distinguishable, while Molt-16 and Jurkat show a similar metabolic profile.

Lipidomic analysis reveals a marked distinction among the four cell lines under study. The absolute amount of intracellular lipids manifests statistically significant differences, with the Molt-4 cell line having a higher amount overall than the other lines. In addition, significant variations are observed at the level of lipid classes. Lipid classes such as PC, PE, PS, PI, and SM manifest differences in at least one of the cell lines, suggesting a possible diversification in the lipid composition of both the cell membrane and the intracellular environment. Such changes in lipid classes may be attributable to changes in the activity of specific enzymes responsible for intracellular lipid synthesis, such as phosphotransferases, phospholipases, phosphatases, lipid kinases, and sphingosine kinases. It is relevant to note that numerous scientific studies have demonstrated activity variation with many of these enzymes in the context of cancer [42,43,44,45]. Interestingly, the percentages of lipid classes in each cell line confirm a uniform distribution of lipid classes across all the cell lines. Our results, based on semiquantitative lipid assessment, support the hypothesis that phospholipid transformation and synthesis pathways diverge among the four cell lines under study. This suggests the possibility of further classification of these tumors based on these lipid classes as biomarkers and even identifying specific pathways that could represent potential therapeutic targets.

Furthermore, specific molecular lipids with statistically significant variations were identified. The heatmap revealed that particular lipid species, such as PE P-16:0_22:3, PE P-18:0_22:4, PE P-18:0_22:3, SM 16:1;O2/26:1, SM 18:1;O2/24:0, PI 18:0_18:1, and PE O-24:2_20:4, show noticeable differences within at least one of the cell lines. These molecules could be the subject of further studies to identify potential biomarkers. In fact, at this level of analysis, the Molt-16 cell line demonstrates a unique lipidomic profile compared to the other cell lines, while Jurkat and Molt-4 share a similar lipid profile. CCRF-CEM also distinguishes itself from the others, albeit to a lesser extent than Molt-16.

Lipid building block analyses revealed significant variations in the levels of fatty acid chains within the lipids of the four cell lines. Specifically, Molt-4 and Molt-16 cells exhibit a relatively higher presence of long-chain fatty acids (with 16–18 carbon atoms) compared to the other two cell lines. Conversely, CCRF-CEM and Molt-16 cells show an increase in lipids with ether-linked chains when compared to Molt-4 and Jurkat lines. These differences suggest distinct activities of enzymes involved in the elongation of fatty acids, such as elongase. While the ratios of SFA/MUFA and SFA/PUFA, calculated to study differences in the unsaturation within the lipid chains, show minimal variations, Molt-16 demonstrates lower ratio values. These differences in lipid-building chains could be associated with higher activity in enzymes regulating these fatty acids, such as specific desaturases.

Studies in different types of tumors have shown an increase in the expression of specific elongases, including elongases 1, 5, and 6 [46]. These enzymes are responsible for elongation of long-chain fatty acids, which include chains of 16, 18, and 20 carbon atoms. Further study showed that elongase 5 plays a key role in lipid metabolism in T cells. This was confirmed by the observation of high expression of this enzyme in proliferating T cells and Jurkat cells compared with resting T cells [47]. The same study also documented increased expression of fatty acid desaturases 1 and 2, which are responsible for the desaturation of long-chain fatty acids.

Our results reveal distinct metabolic behaviors across the four cell lines grown and maintained at the same experimental conditions. Specific pathway shows enhanced activity in one cell line more than the others. This observation not only aids in further classification of these cell lines but also paves the way for in vivo studies to validate these pathways as potential biomarkers. Consequently, these findings could be considered as promising diagnostic biomarkers for T-ALL patients based on the metabolic phenotype. These biochemical features from our preliminary study could then be validated and confirmed in patients. They could then be used to monitor the tumor, track its severity or progression during treatment, and, within specific biological pathways, could also be used to identify new therapeutic targets, thus allowing the assignment of different therapeutic approaches to patients of the same genetic subgroup, based on this classification and stratification due to metabolic phenotype. This encourages a shift towards personalized therapy approaches grounded in metabolic classification to integrate the genetic approach. Moreover, this study could serve as a foundation for additional research exploring the metabolic behavior of T-ALL in other genetic subgroups, as well as those categorized as unclassified due to the absence of cytogenetic markers [10]. Notably, recent studies demonstrated the feasibility of stratifying patients with acute myeloid leukemia (AML) using a lipidomic approach, revealing different lipid profiles among patients with distinct cytogenetic alterations [48]. Nonetheless, it is still necessary to verify and validate these results on primary tumor cells or on samples taken directly from patients in order to translate these findings into potential clinical applications.

## 4. Materials and Methods

### 4.1. Chemicals and Reagents

LC/MS grade water (H_2_O) (Merck KGaA, Darmstadt, Germany) (LiChrosolv), acetonitrile (ACN), methanol (MeOH), isopropanol (IPA), toluene (Tol) all (Carlo Erba, Milan, Italy), ammonium fluoride, ammonium acetate, methyl-t-butyl ether (MTBE), and chloroform (CHCl_3_) were purchased from Sigma-Aldrich (Sigma-Aldrich GmbH, Hamburg, Germany). The internal standard (IS) for lipidomics analysis was EquiSPLASH Lipidomix (Avanti Polar, Alabaster, AL, USA): a mixture with known concentrations of the following lipids [100 μg/mL]: PC 15:0_18:1(d7); PE 15:0_18:1(d7); PS 15:0_18:1(d7); PG 15:0_18:1(d7); PI 15:0_18:1(d7); C15 Ceramide-d7; LPC 18:1(d7); LPE 18:1(d7); CE 18:1(d7); MG 18:1(d7); DG 15:0_18:1(d7); TG 15:0_18:1(d7)_15:0; SM 18:1;2O/18:1(d9); and cholesterol(d7). The cell culture reagents were from Euroclone S.p.A (Pero, Italy).

The Jurkat cell line (TIB-152) was purchased at American Type Culture Collection, Manassas, VA, U.S.A. CCRF-CEM (ACC 240), MOLT-4 (ACC 362), and MOLT-16 (ACC 29) cell lines were kindly provided by the laboratories of Prof. Giovanni Roti (University of Parma, Italy) and purchased at the DSMZ-German Collection of Microorganisms and Cell Cultures GmbH, Germany.

### 4.2. Samples and Sample Preparation

#### 4.2.1. Cell Lines

This study was carried out on four T-acute lymphoblastic leukemia cell lines: Jurkat, CCRF-CEM, MOLT-16, and MOLT-4, all belonging to the TAL/LMO transcriptional complex [49,50], with a well-characterized immunophenotype [30]; see Table S6 for more details. The cell lines were cultured in RPMI 1640 medium with the addition of 10% heat-inactivated fetal bovine serum (FBS) and 1% penicillin/streptomycin (P/S). Cells were kept in the same experimental conditions: in T-75 flasks in a humidified atmosphere containing 5% CO_2_ at a temperature of 37 °C. In order to obtain better results and minimize variations, cells from all four cell lines were seeded at the same concentration and, after 48 h, were harvested, counted, and collected into a pellet via centrifugation at 300× *g*, washed twice with phosphate-buffered saline (PBS), and stored at −80 °C until LC/MS analysis.

#### 4.2.2. Preparation of Analytical Samples and Metabolites Extraction

For polar metabolite analyses, from each cell line, 5 experimental replicates were prepared in 5 different T-75 flasks. After incubation of the cells under physiological conditions for 48 h, 1 million cells were collected, pelleted, and washed twice with PBS. The extraction of polar metabolites was performed following the biphasic method described by Cajka et al. [51], with some minor modifications. A total of 275 µL of MeOH was added to each cell pellet, followed by 30 s of vortexing. Subsequently, 1 mL of MTBE was added, and the sample was again vortexed and shaken for 20 min at 1500 rpm using a T-Shaker (Euroclone). After that, 275 µL of a 10% MeOH solution was added. The sample was centrifuged at 16,000× *g* for 10 min at 4 °C. After phase separation, the polar lower phase was transferred to a glass vial and evaporated with a gentle flow of nitrogen at 60 degrees. The dry residue was resuspended in 100 µL of ACN/H_2_O 4:1 and then placed in the autosampler. The same procedures were followed on a pool of all cells and on an empty Eppendorf tube, which was subsequently used as a blank control.

For the lipidomic analysis, the sample pellet preparation procedure was identical to the previous one. With some modifications, lipid extraction was performed on the pellet following the lipid solubilization process described by Pellegrino et al. [52]. Initially, an extraction mixture was prepared consisting of 33 mL of MTBE, MeOH, and CHCl_3_ at a ratio of 1:1:1 (MMC). The MMC consists of 1 mL of IS diluted 1:10 in MeOH, 10 mL of MeOH, 11 mL of MTBE, and 11 mL of CHCl_3_. Subsequently, 1 mL of the MMC mixture was added to each sample pellet. After 30 s of vortexing, the samples were placed in a T-Shaker (Euroclone) and processed for 20 min at 1500 rpm at 20 °C. Afterward, the samples were centrifuged at 16,000× *g* for 10 min at 4 °C, and the supernatant was transferred to a glass vial. Following this, the supernatant was dried with a light stream of nitrogen. Dried residue was resuspended in 100 µL of MeOH/Tol 9:1 and placed in the autosampler. The same procedures were followed on a pool of all cells and on an Eppendorf containing only the solubilization solution (MMC), which was subsequently used as a blank control.

### 4.3. LC/MS Analysis

LC/MS analysis was conducted using an Agilent system consisting of an Agilent 1260 Infinity II liquid chromatograph consisted of a quaternary pump, a thermostatted column compartment, and an autosampler coupled to an Agilent 6530 Accurate-Mass Q-TOF (Quadrupole-Time-of-Flight) analyzer and an Agilent JetStream source.

#### 4.3.1. Untargeted Polar Metabolomics

Untargeted polar metabolomics chromatographic separation was conducted by following the indications reported by Jian Li et al. [53]. For the immobile phase, a Waters XBridge BEH Amide (HILIC) column (150 mm, 2.1 mm, and 2.5 µm) at 25 °C with a flow rate of 0.35 mL/min was used. The mobile phase consisted of a mixture of water (A) and ACN (B), both with 0.2% formic acid. The gradient used was as follows: From 0 to 3 min, an isocratic gradient was maintained, with A at 10% and B at 90%. From 3 to 13 min, a linear gradient was used, with A at 52% and B at 48%. From 13 to 15 min, an isocratic gradient was maintained, with A at 52% and B at 48%. From 15 to 16 min, a linear gradient was used, with A at 10% and B at 90%. From 16 to 20 min, an isocratic gradient was maintained, with A at 10% and B at 90%. At 20 min, the run was stopped. Spectrometric data were acquired in the range of 40–1700 m/z in both negative and positive polarity. The Agilent JetStream source was used with the following settings: gas temperature (N_2_) at 200 °C, drying gas flow at 10 L/min, nebulizer pressure at 50 psi, and sheath gas temperature at 300 °C with a flow of 12 L/min.

#### 4.3.2. Untargeted Lipidomics

Lipid chromatographic separation was performed as previously described [25,54], employing the same column and conditions: a Supelco Ascentis Express 90A C18 column (100 mm × 2.1 mm, 2.0 µm) maintained at 50 °C with a flow rate of 0.25 mL/min. The mobile phase consisted of four eluents: water, ACN, MeOH, and IPA. All solvents were prepared with ammonium fluoride at a concentration of 0.2 mM. Additionally, IPA, MeOH, and water were prepared with ammonium acetate at a concentration of 10 mM.

In full scan mode, spectrometric data were collected in the range of 50–3200 m/z for both negative and positive polarity. The pool sample was analyzed five times using an iterative DDA acquisition mode to capture as many MS/MS spectra as possible. The Agilent JetStream source settings were consistent with those described in previous works [25,54].

### 4.4. Raw Data Processing

The raw data for metabolic and lipid analysis were processed using MS-DIAL software (version 4.9). This process included peak selection, alignment, and data annotation [55]. In the case of polar metabolites, the annotation was conducted using mass spectra (MS) and mass/tandem spectra (MS/MS) using the NIST2020 database. To conduct the statistical analysis of the polar metabolites, the relative abundances of the metabolites (in terms of areas under the peaks) were measured and normalized according to the number of cells. Subsequently, these data were compared and studied using the MetaboAnalyst platform [19]. The resulting matrix was imported into MetaboAnalyst, where a normalization of the samples was applied with the median. In addition, a logarithmic transformation of the data was performed, followed by pareto scaling. The MetaboAnalyst interface allowed the execution of various analyses, including principal component analysis (PCA), one-way ANOVA tests, and the creation of a hierarchical clustering dendrogram. In addition, the possibility was given to conduct pathway analyses to compare variations in biochemical pathways between different cell lines. These pathways and the enzymes involved are based on the Homo Sapiens KEGG databases.

For lipid annotation, following the guidelines dictated by the Lipid Standard Initiative (LSI), the annotation of lipid molecular species was performed at the molecular level using data acquired with mass spectrometry (MS) and tandem mass spectrometry (MS/MS) [56] using databases generated through the Lipid Spectrum Generator (LSG) tool [57]. Following the procedure outlined by Tsugawa H. et al. [55], lipid semiquantification was performed using the deuterated internal standard (EquiSPLASH Lipidomix) for each lipid class at levels 2 and 3 of the LSI recommendations (see Table S7). Semiquantification of the raw data was conducted using an R script developed within our laboratory. The semiquantification was based on the known concentrations of the standards added to the samples. At the end of this analytical step, a set of matrices was produced containing the expressive concentrations in µg/1 million cells of the lipid molecular species previously annotated and classified according to the different lipid classes. These matrices were subjected to median normalization and then pareto scaling was applied, helping to prepare the final matrix for subsequent analyses. Statistical analyses on the identified variations within the lipid profiles were performed using a one-way ANOVA test, PCA, and heatmap visualization. Specifically, the matrix of lipid concentrations was examined using LipidOne [21], supported with R scripts developed in-house to generate visualizations such as the PCA score graph, heatmap, and bar graphs.

## 5. Conclusions

In this study, metabolomics and lipidomics analyses of Jurkat, Molt-16, Molt-4, and CCRF-CEM cell lines allowed the annotation of 126 polar metabolites and 217 different lipid species.

The experimental data confirm the possibility to differentiate T-cell acute lymphoblastic leukemia cell lines belonging to the same genetic subgroup. Notably, when considering the overall metabolic profile, it becomes apparent that Molt-4 stands out as the most distinct among the cell lines. However, digging into the detailed pathway and lipidomic analysis allows for the differentiation and stratification of these cell lines based on specific pathways. For example, CCRF-CEM is distinguishable with its high-activity in purine metabolism, Molt-4 is distinguishable with its unique behavior regarding glutathione metabolism and acetylcholine, or the unique lipidomic profile of Molt-16. On the other hand, the results also show some similarities among the cell lines. The overall metabolic and lipidic profiles indicate a resemblance between Jurkat and Molt-16. Furthermore, these cell lines share similar behaviors in specific metabolic pathways, such as purine metabolism and glutathione metabolism.

Consequently, our study introduces the potential for an additional subclassification for T-ALL based on the metabolic and lipid phenotype. The identified differences among the four cell lines call for a more in-depth investigation utilizing cells directly sourced from patients with TAL/LMO rearrangements to validate these observed distinctions as metabolic biomarkers. Such biochemical features could be used to classify patients into multiple groups based on metabolomic profile. This could be useful at a clinical level for diagnosis, as well as for stratification of disease severity and risk. Furthermore, they could be used to monitor disease progression during specific treatments. Ultimately, this classification could be useful for the discovery of potential therapies, based on the altered metabolic pathways within patients in each metabolomic subclass.

Moreover, within the context of T-ALL, cases labeled as ‘unclassified’ due to the absence of cytogenetic markers could benefit from multiomics approaches, such as metabolomics and lipidomics. This could unveil underlying leukemogenic mechanisms, leading to the identification of new biomarkers specific to each tumor variant. Such insights could facilitate the identification of tailored therapeutic targets based on the metabolic behavior of each tumor variant.

In conclusion, this study demonstrates the potential of the metabolomic and lipidomic approach as valuable tools offering a comprehensive view of the cells and their progression. This opens the possibility of designing experiments to directly verify and confirm the obtained results in patients.

## Figures and Tables

**Table 1:** Comparison of the concentration of lipid classes of different T-cell lines; the number of replicates is 5. The average concentrations are reported in ug/10^6^ cells. The number of annotated molecules is the number of single molecular species revealed.

Lipid Class Abbreviation	Explained Class Name	Number of Annotated Molecules	Average ± Exp Er (Jurkat)	Average ± Exp Er (CCRF-CEM)	Average ± Exp Er (MOLT-4)	Average ± Exp Er (MOLT-16)	*p*-Value
CE	Cholesteryl ester	6	0.54559 (±0.07061)	0.89815 (±0.06599)	0.69316 (±0.07075)	1.13462 (±0.12362)	0.001093
CL	Cardiolipin	9	0.03421 (±0.00316)	0.03732 (±0.00131)	0.04399 (±0.00265)	0.02556 (±0.00065)	0.000741
Cer	Ceramide	5	0.0371 (±0.00355)	0.04498 (±0.00264)	0.07195 (±0.01037)	0.08302 (±0.00841)	0.000205
DG	Diacylglycerol	9	0.08151 (±0.00735)	0.07011 (±0.00243)	0.10137 (±0.00789)	0.09266 (±0.00154)	0.006469
HexCer	Hexosylceramide	2	0.00399 (±0.00049)	0.00274 (±0.00011)	0.00754 (±0.00154)	0.00554 (±0.0003)	0.004946
LPC	Lysophophatidylcholine	3	0.05673 (±0.01429)	0.03656 (±0.00711)	0.04768 (±0.00934)	0.08803 (±0.02083)	0.094575
LPE	Lysophosphatidylethanolamine	1	0.09433 (±0.05043)	0.03227 (±0.00886)	0.0462 (±0.00317)	0.0435 (±0.01015)	0.37543
PC	Phosphatidylcholine	42	3.46958 (±0.21436)	3.36706 (±0.03942)	4.40161 (±0.06745)	4.39338 (±0.07721)	6.1 × $10^{-6}$
PC-O	Alkyl Ether-linked phosphatidylcholine	10	0.59456 (±0.06462)	1.03528 (±0.03938)	1.25128 (±0.08139)	0.58819 (±0.05143)	1.01 × $10^{-6}$
PC-P	Vinyl Ether-linked phosphatidylcholine	5	0.10567 (±0.01139)	0.08346 (±0.00222)	0.16357 (±0.00725)	0.05577 (±0.00151)	5.72 × $10^{-8}$
PE	Phosphatidylethanolamine	20	0.92424 (±0.0747)	0.86159 (±0.03958)	1.20771 (±0.03715)	1.2668 (±0.02376)	2.27 × $10^{-5}$
PE-O	Alkyl Ether-linked phosphatidylethanolamine	8	0.21023 (±0.02108)	0.23111 (±0.00425)	0.28497 (±0.0164)	0.15191 (±0.0111)	9.12 × $10^{-5}$
PE-P	Vinyl Ether-linked phosphatidylethanolamine	18	0.71301 (±0.06548)	0.88215 (±0.02568)	1.06558 (±0.04709)	0.49967 (±0.02099)	7.29 × $10^{-7}$
PG	Phosphatidylglycerol	2	0.04741 (±0.00487)	0.0251 (±0.00093)	0.07804 (±0.00477)	0.04413 (±0.00145)	1.42 × $10^{-7}$
PI	Phosphatidylinositol	15	0.70036 (±0.08017)	0.83635 (±0.01766)	0.96282 (±0.04508)	0.87907 (±0.03204)	0.013254
PI-O	Ether-linked phosphatidylinositol	1	0.00489 (±0.00067)	0.00513 (±0.0003)	0.00523 (±0.00033)	0.00634 (±0.00061)	0.22212
PS	Phosphatidylserine	10	0.65673 (±0.05325)	0.70552 (±0.01925)	0.68516 (±0.01973)	0.79491 (±0.02234)	0.040757
SM	Sphingomyelin	9	0.65778 (±0.03399)	0.58726 (±0.01193)	0.87832(±0.04014)	0.80118 (±0.02078)	8.53 × $10^{-6}$
TG	Triacylglycerol	42	0.12245 (±0.00637)	0.19821 (±0.0283)	0.73949 (±0.07466)	0.19183 (±0.02192)	3.39 × $10^{-8}$
Total		217	9.06037 (±0.4956)	9.94036 (±0.15381)	12.73568 (±0.21066)	11.14609 (±0.13639)	7.45 × $10^{-7}$

**Table 2:** Ratio of saturated fatty acids (SFA), monounsaturated fatty acids (MUFA), and polyunsaturated fatty acids (PUFA) using the average amount of chains with different types of saturations (n = 5) in μg/10^6^ of cells.

Cell Line/Ratio	SFA/MUFA	SFA/PUFA
Jurkat	0.9393	1.7644
CCRF-CEM	0.9926	1.8439
Molt-4	0.9765	2.1925
Molt-16	0.8479	1.4532

## Data Availability

The datasets generated during and/or analyzed during the current study are available from the corresponding author on reasonable request.

## Supplementary Materials

The following supporting information can be downloaded at: https://www.mdpi.com/article/10.3390/ijms25073921/s1.

## References

[1] Van Vlierberghe P., Ferrando A. (2012). The Molecular Basis of T Cell Acute Lymphoblastic Leukemia. J. Clin. Investig..

[2] Litzow M.R., Ferrando A.A. (2015). How I Treat T-Cell Acute Lymphoblastic Leukemia in Adults. Blood.

[3] Karrman K., Johansson B. (2017). Pediatric T-cell Acute Lymphoblastic Leukemia. Genes Chromos. Cancer.

[4] Chiaretti S., Foà R. (2009). T-Cell Acute Lymphoblastic Leukemia. Haematologica.

[5] Puckett Y., Chan O. (2023). Acute Lymphocytic Leukemia.

[6] Ferrando A.A., Look A.T. (2003). Gene Expression Profiling in T-Cell Acute Lymphoblastic Leukemia. Semin. Hematol..

[7] La Starza R., Pierini V., Pierini T., Nofrini V., Matteucci C., Arniani S., Moretti M., Lema Fernandez A.G., Pellanera F., Di Giacomo D. (2020). Design of a Comprehensive Fluorescence in Situ Hybridization Assay for Genetic Classification of T-Cell Acute Lymphoblastic Leukemia. J. Mol. Diagn..

[8] Atak Z.K., Gianfelici V., Hulselmans G., De Keersmaecker K., Devasia A.G., Geerdens E., Mentens N., Chiaretti S., Durinck K., Uyttebroeck A. (2013). Comprehensive Analysis of Transcriptome Variation Uncovers Known and Novel Driver Events in T-Cell Acute Lymphoblastic Leukemia. PLoS Genet..

[9] Franciosa G., Smits J.G.A., Minuzzo S., Martinez-Val A., Indraccolo S., Olsen J. (2021). V Proteomics of Resistance to Notch1 Inhibition in Acute Lymphoblastic Leukemia Reveals Targetable Kinase Signatures. Nat. Commun..

[10] Bardelli V., Arniani S., Pierini V., Di Giacomo D., Pierini T., Gorello P., Mecucci C., La Starza R. (2021). T-Cell Acute Lymphoblastic Leukemia: Biomarkers and Their Clinical Usefulness. Genes.

[11] Van Vlierberghe P., Pieters R., Beverloo H.B., Meijerink J.P.P. (2008). Molecular-Genetic Insights in Paediatric T-Cell Acute Lymphoblastic Leukaemia. Br. J. Haematol..

[12] Lima A.R., Carvalho M., Aveiro S.S., Melo T., Domingues M.R., Macedo-Silva C., Coimbra N., Jerónimo C., Henrique R., Bastos M.D.L. (2022). Comprehensive Metabolomics and Lipidomics Profiling of Prostate Cancer Tissue Reveals Metabolic Dysregulations Associated with Disease Development. J. Proteome Res..

[13] Armitage E.G., Southam A.D. (2016). Monitoring Cancer Prognosis, Diagnosis and Treatment Efficacy Using Metabolomics and Lipidomics. Metabolomics.

[14] Wang R., Li B., Lam S.M., Shui G. (2020). Integration of Lipidomics and Metabolomics for In-Depth Understanding of Cellular Mechanism and Disease Progression. J. Genet. Genom..

[15] Wang J., Wang C., Han X. (2019). Tutorial on Lipidomics. Anal. Chim. Acta.

[16] Hollywood K., Brison D.R., Goodacre R. (2006). Metabolomics: Current Technologies and Future Trends. Proteomics.

[17] Züllig T., Trötzmüller M., Köfeler H.C. (2020). Lipidomics from Sample Preparation to Data Analysis: A Primer. Anal. Bioanal. Chem..

[18] Harrieder E.-M., Kretschmer F., Böcker S., Witting M. (2022). Current State-of-the-Art of Separation Methods Used in LC-MS Based Metabolomics and Lipidomics. J. Chromatogr. B Analyt. Technol. Biomed. Life Sci..

[19] Pang Z., Chong J., Zhou G., de Lima Morais D.A., Chang L., Barrette M., Gauthier C., Jacques P.-É., Li S., Xia J. (2021). MetaboAnalyst 5.0: Narrowing the Gap between Raw Spectra and Functional Insights. Nucleic Acids Res..

[20] Zhou G., Pang Z., Lu Y., Ewald J., Xia J. (2022). OmicsNet 2.0: A Web-Based Platform for Multi-Omics Integration and Network Visual Analytics. Nucleic Acids Res..

[21] Pellegrino R.M., Giulietti M., Alabed H.B.R., Buratta S., Urbanelli L., Piva F., Emiliani C. (2022). LipidOne: User-Friendly Lipidomic Data Analysis Tool for a Deeper Interpretation in a Systems Biology Scenario. Bioinformatics.

[22] Gaud C., Sousa B.C., Nguyen A., Fedorova M., Ni Z., O’Donnell V.B., Wakelam M.J.O., Andrews S., Lopez-Clavijo A.F. (2021). BioPAN: A Web-Based Tool to Explore Mammalian Lipidome Metabolic Pathways on LIPID MAPS. F1000Research.

[23] Park J.Y., Lee S.-H., Shin M.-J., Hwang G.-S. (2015). Alteration in Metabolic Signature and Lipid Metabolism in Patients with Angina Pectoris and Myocardial Infarction. PLoS ONE.

[24] Kohno S., Keenan A.L., Ntambi J.M., Miyazaki M. (2018). Lipidomic Insight into Cardiovascular Diseases. Biochem. Biophys. Res. Commun..

[25] Alabed H.B.R., Gorello P., Pellegrino R.M., Lancioni H., La Starza R., Taddei A.A., Urbanelli L., Buratta S., Fernandez A.G.L., Matteucci C. (2023). Comparison between Sickle Cell Disease Patients and Healthy Donors: Untargeted Lipidomic Study of Erythrocytes. Int. J. Mol. Sci..

[26] Wang Y.-N., Ma S.-X., Chen Y.-Y., Chen L., Liu B.-L., Liu Q.-Q., Zhao Y.-Y. (2019). Chronic Kidney Disease: Biomarker Diagnosis to Therapeutic Targets. Clin. Chim. Acta.

[27] Wood P.L. (2014). Mass Spectrometry Strategies for Clinical Metabolomics and Lipidomics in Psychiatry, Neurology, and Neuro-Oncology. Neuropsychopharmacology.

[28] Li Y., Liang L., Deng X., Zhong L. (2019). Lipidomic and Metabolomic Profiling Reveals Novel Candidate Biomarkers in Active Systemic Lupus Erythematosus. Int. J. Clin. Exp. Pathol..

[29] Farrokhi Yekta R., Rezaie Tavirani M., Arefi Oskouie A., Mohajeri-Tehrani M.R., Soroush A.R. (2016). The Metabolomics and Lipidomics Window into Thyroid Cancer Research. Biomarkers.

[30] Burger R., Hansen-Hagge T.E., Drexler H.G., Gramatzki M. (1999). Heterogeneity of T-Acute Lymphoblastic Leukemia (T-ALL) Cell Lines: Suggestion for Classification by Immunophenotype and T-Cell Receptor Studies. Leuk. Res..

[31] Alarcon-Barrera J.C., von Hegedus J.H., Brouwers H., Steenvoorden E., Ioan-Facsinay A., Mayboroda O.A., Ondo-Mendez A., Giera M. (2020). Lipid Metabolism of Leukocytes in the Unstimulated and Activated States. Anal. Bioanal. Chem..

[32] Kwon H.-Y., Kumar Das R., Jung G.T., Lee H.-G., Lee S.H., Berry S.N., Tan J.K.S., Park S., Yang J.-S., Park S. (2021). Lipid-Oriented Live-Cell Distinction of B and T Lymphocytes. J. Am. Chem. Soc..

[33] van Baren N., Van den Eynde B.J. (2015). Tryptophan-Degrading Enzymes in Tumoral Immune Resistance. Front. Immunol..

[34] Lu S.C. (2013). Glutathione Synthesis. Biochim. Biophys. Acta (BBA) Gen. Subj..

[35] Bansal A., Simon M.C. (2018). Glutathione Metabolism in Cancer Progression and Treatment Resistance. J. Cell Biol..

[36] Cheng C.-T., Qi Y., Wang Y.-C., Chi K.K., Chung Y., Ouyang C., Chen Y.-R., Oh M.E., Sheng X., Tang Y. (2018). Arginine Starvation Kills Tumor Cells through Aspartate Exhaustion and Mitochondrial Dysfunction. Commun. Biol..

[37] Yin J., Ren W., Huang X., Deng J., Li T., Yin Y. (2018). Potential Mechanisms Connecting Purine Metabolism and Cancer Therapy. Front. Immunol..

[38] Barfeld S.J., Fazli L., Persson M., Marjavaara L., Urbanucci A., Kaukoniemi K.M., Rennie P.S., Ceder Y., Chabes A., Visakorpi T. (2015). Myc-Dependent Purine Biosynthesis Affects Nucleolar Stress and Therapy Response in Prostate Cancer. Oncotarget.

[39] Goswami M.T., Chen G., Chakravarthi B.V.S.K., Pathi S.S., Anand S.K., Carskadon S.L., Giordano T.J., Chinnaiyan A.M., Thomas D.G., Palanisamy N. (2015). Role and Regulation of Coordinately Expressed de Novo Purine Biosynthetic Enzymes PPAT and PAICS in Lung Cancer. Oncotarget.

[40] Bahreyni A., Samani S.S., Rahmani F., Behnam-Rassouli R., Khazaei M., Ryzhikov M., Parizadeh M.R., Avan A., Hassanian S.M. (2018). Role of Adenosine Signaling in the Pathogenesis of Breast Cancer. J. Cell Physiol..

[41] Yamauchi T., Miyawaki K., Semba Y., Takahashi M., Izumi Y., Nogami J., Nakao F., Sugio T., Sasaki K., Pinello L. (2022). Targeting Leukemia-Specific Dependence on the de Novo Purine Synthesis Pathway. Leukemia.

[42] Meana C., García-Rostán G., Peña L., Lordén G., Cubero Á., Orduña A., Győrffy B., Balsinde J., Balboa M.A. (2018). The Phosphatidic Acid Phosphatase Lipin-1 Facilitates Inflammation-Driven Colon Carcinogenesis. JCI Insight.

[43] He J., Zhang F., Tay L.W.R., Boroda S., Nian W., Levental K.R., Levental I., Harris T.E., Chang J.T., Du G. (2017). Lipin-1 Regulation of Phospholipid Synthesis Maintains Endoplasmic Reticulum Homeostasis and Is Critical for Triple-Negative Breast Cancer Cell Survival. FASEB J..

[44] Sanchez-Lopez E., Zimmerman T., Gomez del Pulgar T., Moyer M.P., Lacal Sanjuan J.C., Cebrian A. (2013). Choline Kinase Inhibition Induces Exacerbated Endoplasmic Reticulum Stress and Triggers Apoptosis via CHOP in Cancer Cells. Cell Death Dis..

[45] Arora G.K., Palamiuc L., Emerling B.M. (2022). Expanding Role of PI5P4Ks in Cancer: A Promising Druggable Target. FEBS Lett..

[46] Yamashita Y., Nishiumi S., Kono S., Takao S., Azuma T., Yoshida M. (2017). Differences in Elongation of Very Long Chain Fatty Acids and Fatty Acid Metabolism between Triple-Negative and Hormone Receptor-Positive Breast Cancer. BMC Cancer.

[47] Robichaud P.-P., Munganyiki J.E., Boilard E., Surette M.E. (2018). Polyunsaturated Fatty Acid Elongation and Desaturation in Activated Human T-Cells: ELOVL5 Is the Key Elongase. J. Lipid Res..

[48] Stefanko A., Thiede C., Ehninger G., Simons K., Grzybek M. (2017). Lipidomic Approach for Stratification of Acute Myeloid Leukemia Patients. PLoS ONE.

[49] Sanda T., Lawton L.N., Barrasa M.I., Fan Z.P., Kohlhammer H., Gutierrez A., Ma W., Tatarek J., Ahn Y., Kelliher M.A. (2012). Core Transcriptional Regulatory Circuit Controlled by the TAL1 Complex in Human T Cell Acute Lymphoblastic Leukemia. Cancer Cell.

[50] Sharma A., Mistriel-Zerbib S., Najar R.A., Engal E., Bentata M., Taqatqa N., Dahan S., Cohen K., Jaffe-Herman S., Geminder O. (2023). Isoforms of the TAL1 Transcription Factor Have Different Roles in Hematopoiesis and Cell Growth. PLoS Biol..

[51] Cajka T., Hricko J., Rudl Kulhava L., Paucova M., Novakova M., Kuda O. (2023). Optimization of Mobile Phase Modifiers for Fast LC-MS-Based Untargeted Metabolomics and Lipidomics. Int. J. Mol. Sci..

[52] Pellegrino R.M., Di Veroli A., Valeri A., Goracci L., Cruciani G. (2014). LC/MS Lipid Profiling from Human Serum: A New Method for Global Lipid Extraction. Anal. Bioanal. Chem..

[53] Li J., Ma J., Li Q., Fan S., Fan L., Ma H., Zhang Y., Zheng L. (2021). Determination of 35 Free Amino Acids in Tea Using Ultra-Performance Liquid Chromatography Coupled with Quadrupole Time-of-Flight Mass Spectrometry. Front. Nutr..

[54] Alabed H.B.R., Del Grosso A., Bellani V., Urbanelli L., Carpi S., De Sarlo M., Bertocci L., Colagiorgio L., Buratta S., Scaccini L. (2023). Untargeted Lipidomic Approach for Studying Different Nervous System Tissues of the Murine Model of Krabbe Disease. Biomolecules.

[55] Tsugawa H., Ikeda K., Takahashi M., Satoh A., Mori Y., Uchino H., Okahashi N., Yamada Y., Tada I., Bonini P. (2020). A Lipidome Atlas in MS-DIAL 4. Nat. Biotechnol..

[56] Liebisch G., Fahy E., Aoki J., Dennis E.A., Durand T., Ejsing C.S., Fedorova M., Feussner I., Griffiths W.J., Köfeler H. (2020). Update on LIPID MAPS Classification, Nomenclature, and Shorthand Notation for MS-Derived Lipid Structures. J. Lipid Res..

[57] Gertner D.S., Violi J.P., Bishop D.P., Padula M.P. (2023). Lipid Spectrum Generator: A Simple Script for the Generation of Accurate In Silico Lipid Fragmentation Spectra. Anal. Chem..

